# Holistic rehabilitation for children with cancer: The Chilean model

**DOI:** 10.1002/cnr2.1515

**Published:** 2021-07-26

**Authors:** Vera Celedón, Nuria Rossell, Marcela Zubieta

**Affiliations:** ^1^ Fundación Nuestros Hijos Santiago Chile

**Keywords:** childhood cancer, Chile, rehabilitation

## Abstract

The increasingly positive outcomes of childhood cancer treatments are among the most inspiring stories in modern medicine. Many of the children and adolescents surviving cancer will have a healthy life; however, many others will suffer from physical, cognitive, psychological, and social sequelae. During treatment, many children experience multiple temporary and permanent side effects which negatively impact their quality of life. Low‐ and middle‐income countries where childhood cancer treatment outcomes are improving are facing the reality of a growing population of teenagers and young adults suffering from long‐term disease‐ and treatment‐related consequences. In Chile, 500 children are diagnosed with cancer each year. Treatment is granted for all through public health policies and NGO collaboration. In order to address the complex problems from acute and long‐term consequences of disease and treatment, the Oncological Rehabilitation Center Fundación Nuestros Hijos (CROFNH) provides multidisciplinary attention to an extensive variety of rehabilitation needs for children and adolescents with cancer. With its integrated services in the medical treatment of children and adolescents with cancer, the CROFNH helps reduce the impact of treatment‐related side effects in children's daily lives, improves quality of life, and aims at contributing to these children becoming independent and functional adults to the maximum of their capacities. The aim of this article is to show the experience of the Chilean Oncological Rehabilitation Centre and its unique multidisciplinary approach. In addition, we discuss the successful telerehabilitation strategy implemented in response to the COVID‐19 pandemic in order to secure continuity of treatment.

## 1 BACKGROUND

Childhood cancer is a disease of low incidence, high complexity, and biopsychosocial impact.^1^, ^2^, ^3^, ^4^, ^5^, ^6^ Around 300 000 people between 0 and 19 years of age are diagnosed each year; that is, 822 boys, girls, and adolescents daily.^6^ One‐third of the diagnoses are leukemias, followed by central nervous system tumors and lymphomas.^1^, ^6^, ^7^ The survival rate varies according to the income level of the countries: children with cancer living in low‐ and middle‐income countries have around 20% survival chances, while those living in high‐income countries have up to 80% survival rates.^5^, ^8^, ^9^, ^10^

Increased survival in childhood cancer thanks to more effective but intense treatments is accompanied by physical, cognitive, psychological, and social sequelae, all of which can appear years after the end of treatment.^11^, ^12^, ^13^ These sequelae have an impact on the quality of life (QOL) of the children, adolescents, and their families, altering their functionality, school performance, and social and labor inclusion.^9^, ^13^, ^14^, ^15^ Indeed, cancer survivors have been shown to be 43% less likely to graduate from high school, have 60% less chance of being employed, and 18% less chance of marrying.^14^ Among the physical conditions are fatigue related to cancer, pain, cardiorespiratory dysfunctions, disorders in psychomotor development, osteonecrosis, osteoporosis, amputations, visual alterations, coordination problems, oral infections, mucositis, xerostomia, dental agenesis, malocclusion, disorders of the speech and language, swallowing disorders, and malnutrition due to excess and/or deficiency.^16^, ^17^, ^18^, ^19^, ^20^, ^21^, ^22^, ^23^, ^24^ As part of the cognitive sequelae that affect development, learning, and functionality, the following have been documented: loss of brain volume, brain parenchymal atrophy, leukoencephalopathy, alteration in attention, processing speed, working memory, and visual‐motor integration.^11^, ^12^, ^13^, ^15^, ^24^, ^25^ Among the psychological sequelae, depression, anxiety, social maladjustment, stress, and post‐traumatic stress are documented.^15^, ^24^, ^26^ At an educational level, a childhood cancer survivor is more likely to repeat or not be able to advance to tertiary education, and they also have special educational needs; therefore, they require schools with special support capacity.^24^, ^27^ Due to the multiple temporary and permanent sequelae that a child with cancer can experience, it is necessary to give access to a rehabilitation process, so that in the future they become as independent and functional adults as possible.

However, comprehensive rehabilitation programs for children with cancer and childhood cancer survivors are not common, nor are these programs integrated into health systems in most countries. In the United States, services available for specific therapy needs for cancer patients are offered mainly in big comprehensive cancer centers for adults,^28^ and it has been recognized that rehabilitation services lack integration in tertiary centers and cancer programs.^29^

### 1.1 Childhood cancer in Chile

In Chile, cancer is a public health problem and has been decreed as one of the Government's foci for action.^3^, ^4^ Yearly, 500 cases of childhood cancer are diagnosed, affecting mainly males (55.6%) and the age group under five years.^3^, ^4^, ^30^ The five‐year survival rate has reached an auspicious 78%, approaching the figures of high‐income countries, a fact that is attributed to the public health insurance (FONASA) that serves 78% of the population.^30^, ^31^ These good results are due to multiple actions based on public policies and integrated work: treatment for all children with cancer diagnosed under 15 years of age is covered by explicit health guarantees which regulate provision, access to treatments, and costs; implementation of treatment protocols from developed countries are adapted to the Chilean population; tripartite work exists between the State (MINSAL), NGO's, and the private sector; and the national registry of childhood cancer is based on a legislative framework.^1^, ^3^, ^4^, ^30^ This set of actions has made high‐quality treatment available for all children and adolescents with cancer, but it also enables the coverage of other needs, such as rehabilitation services that improve the children's treatment process and their future quality of life. This article aims to show the experience of the Oncological Rehabilitation Center in Chile (CROFNH) as an example that could inspire replication and collaboration in other countries of the region and beyond. Additionally, we hope to raise awareness of the importance of securing access to specialized rehabilitation for all children and adolescents with cancer.

## 2 ONCOLOGICAL REHABILITATION CENTER FUNDACIÓN NUESTROS HIJOS (CROFNH)

In September 2014, Fundación Nuestros Hijos, an NGO devoted to improving childhood cancer care in Chile, created the Cancer Rehabilitation Center (CROFNH) in order to fill an existing health gap in the country. Both the public and private systems could not ensure access or the opportunity to a specialized, continuous, and intensive rehabilitation process for Chilean oncological children and adolescents. To our knowledge, the CROFNH is the only oncological rehabilitation center in Latin America. Its objective is to provide comprehensive, timely, safe, and quality rehabilitation to children undergoing cancer treatment from cancer's earliest stages until discharge or death.

The CROFNH rehabilitation services are free of cost for patients coming from the public health system (where 80% of the children with cancer are treated).^30^ Those coming from the private system pay a small percentage of the cost, which private insurers reimburse partially. The services are adapted to the needs and circumstances of the children, which means that the CROFNH staff attends in the bed unit of public hospitals (both in the oncology unit and intensive care unit when requested), at the patient's home (ambulatory when the child is unable to attend the center, either due to advanced disease or the use of equipment such as a mechanical ventilator), and in the CROFNH's own facilities located in Santiago, the capital city.

### 2.1 Facilities and operations

The center consists of 1100 m^2^ which are distributed in the areas described in Table 1. To get enrolled in the CROFNH's services, patients are referred by the treating pediatric oncologists from the health center of origin with a referral that includes an updated clinical summary. Within 72 hours from the referral, an admission appointment with a physiatrist is scheduled, who after proper evaluation indicates the specialties that will attend the patient and the frequency of visits. All care is based on a comprehensive evaluation from each specialty involved, which takes into consideration family and clinical history as well as standardized evaluations according to each area. After this, work objectives are proposed and treatment begins, which is evaluated periodically by comparing the results with the baseline level of admission and the previous evaluation. The appointments duration ranges from 45 to 60 minutes, mainly in individual encounters and in some cases integrated (two professionals with a patient). Cross‐cutting therapeutic objectives are worked on and personalized rehabilitation plans are carried out. The clinical team meets once a week for case discussions, unification of criteria, evaluate progress and goals, and jointly discussing solutions for the difficulties detected.

**TABLE 1:** Description of clinical areas at Chile's Oncological Rehabilitation Centre Fundación Nuestros Hijos

Area	Description
Dental box	Equipped with a special couch suitable for attending patients from their wheelchair. Designed to prevent cross infections for attending children and adolescents, as well as caregivers.
Clinical consulting rooms	Nine rooms with basic equipment to provide multidisciplinary care, such as a computer, stretcher, small tables and chairs, and sinks, among others. Each room is decorated with motifs from Chilean flora and fauna.
Therapeutic gym	A spacious and bright space that allows for rehabilitation from the wide range of physical sequelae, improving movement and functionality. Equipped with THERA‐Trainer balo, MOTOmed, anti‐gravity treadmill, vibrating platform, stationary bike, and climbing wall, among other things.
Early care room	A room enabled to work on physical, cognitive, and social skills in children under five years of age. All the equipment is adapted to children's size: kitchen, washing machine, supermarket trolley, sink, crib, and mats, among others.
Room of daily living activities	A room for training activities of daily living, it is a simulation of a home. It has a microwave, electric oven, sofa‐bed, toothpaste dispenser, mirror, desk, computer, closet, hangers, and iron, among other things.
Multisensory stimulation room	Designed for the stimulation of all the senses: auditory, visual, tactile, vestibular and proprioception. It has fiber optics, a water bed, bubble tube, a 52‐inch touch screen, projector, starry sky, tablet, sound, and led lights in the ceiling.
Basic emergency room	A room prepared to provide basic assistance to a patient experiencing a vital emergency while waiting for the ambulance to arrive. Equipped with a wheeled stretcher, AED, portable oxygen, manual resuscitation bag, and secretion aspiration machine, among other things.
Hydrotherapy room	Equipped with a 1200‐liter Hubbard tank, lift and water stretcher, this room is used to carry out therapy in warm water and to improve movements while reducing pain. The water is discarded after each use for biosecurity.
Sensory integration room	It has tunnels, a vestibulator with different swings, stairs, and psychomotor elements (cubes, wedges, cylinders) that allow circuits to work on sensory integration, balance, and motor skills, among other things.
Orthotic room	A room where occupational therapists make resting orthotics, orthopedic insoles, and various technical aids that children and adolescents require to progress in their rehabilitation process. It has a portable and fixed oven, a sewing machine, a deep sink and various tools.
Virtual rehabilitation room	This room has different video game consoles and their respective body reading platforms (Wii, Xbox, Play Station, Nintendo Switch), as well as virtual reality glasses. It allows for working on different objectives: coordination, muscle strengthening, balance, memory, concentration, and mirroring, among other things. By allowing up to four users to work at the same time, it improves social skills and fosters frustration tolerance through play.

In order to comprehensively address the children's needs and promote their social inclusion, since 2016 the CROFNH offers group workshops to work on specific therapeutic objectives combined with social skills. The workshops are addressed to the children about activities like cooking, infant massage (with caregivers), communication, transition to the school stage, use of public transport, visits to museums, and zoos, among others.

In the area of palliative care, the focus of the CROFNH is to take care of the quality of life of children and adolescents by prolonging independence for as long as possible, reducing pain, maintaining comfort, educating the caregiver regarding the progression of symptoms and sequelae as well as training them in its treatment, recognizing signs of deterioration, and signs to expect prior to decease.

### 2.2 Existing specialties

The varied needs of the children are covered by a wide range of specialties: kinesiology, occupational therapy, neuropsychology, speech therapy, physiatry, differentiated education, nutrition, psycho‐oncology, and pediatric dentistry.

In order to achieve proper coordination of the clinical and social aspects of the CROFNH comprehensive, multidisciplinary services, the integrated work of social work and nursing professionals is essential. The social worker assesses the parental skills necessary to keep treatment adherence and generates support networks for patients and their families in order to alleviate, solve, or prevent social problems that may affect the cancer rehabilitation process. In addition, he evaluates the social and housing conditions of patients, identifying the improvements needed to facilitate the child's independence and to incorporate housing changes that support a healthy physical environment. Based on the social report as the main tool that illustrates the social situation of the child and his/her family, various support interventions can be organized. On the other hand, the nurse is in charge of ensuring the quality of the rehabilitation process, the compliance of the process with corresponding health regulations, and the biosafety standards according to the needs of immunocompromised patients.

### 2.3 Patients attended and user satisfaction

**FIGURE 1:**
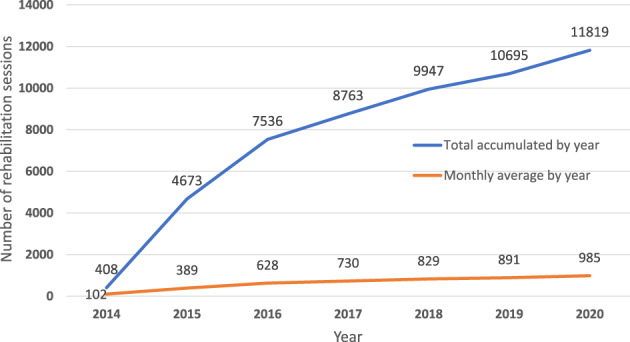
Number of rehabilitation sessions attended by patients at Chile's Oncological Rehabilitation Centre Fundación Nuestros Hijos from September 2014 to December 2020

According to internal data from September 2014 to December 2020, the CROFNH has granted 53 481 services to more than 480 different patients. The patients' age on admission ranged from 23 days to 23 years and the stages of cancer treatment were 79% active treatment, 17% follow‐up, and 4% palliative care. The increase in the number of sessions provided from 2014 to 2020 is shown in Figure 1.

A study about the clinical profile of the children and adolescents attended at the CROFNH from 2014 to 2017 showed that the patients had an average age of 8.7 years upon admission. The most frequent diagnosis was tumors of the central nervous system. Of the total number of patients, 53.2% were undergoing active treatment. Motor deficits were the most frequently diagnosed (79.8%), including hemiparesis, tetraparesis, and fatigue; 28.4% of patients presented swallowing disorders; 34.9% presented visual disorders; and 56.9% presented a cognitive compromise.^32^ The foregoing reinforces the need for a multidisciplinary team to provide care for these patients. Table 2 shows the percentages of patient care by each of the CROFNH subspecialties provided during 2020 by ranking.

**TABLE 2:** Rehabilitation sessions provided by each specialty at Chile's Oncological Rehabilitation Centre Fundación Nuestros Hijos during 2020

Specialty	%
Kinesiology	25.4
Occupational therapist	19.6
Neuropsychology	16.4
Speech therapy	16.4
Physiatry	8.7
Special education	7.7
Odontology	2.1
Pediatric dentistry	1.2
Nutrition	1.0
Psychology	1.0
Social worker	0.5

A user satisfaction survey in 2019 showed that most of the parents of the patients attended in the CROFNH consider that their children like to attend their therapies (92%). All the respondents had positive comments about the CROFNH services and staff, for example, staff being friendly, kind, professional, dedicated, and committed; the service was considered of good quality; and therapy attention was well‐implemented, with good results. Some of the parents comments were as follows: “(The center) is incredible in the human and material aspects”, “is very good at rehabilitating children”, and “provides a lot of support for the child and the family”.

## 3 ADAPTING TO THE SPECIAL CIRCUMSTANCES OF THE COVID‐19 PANDEMIC

The continuity of a rehabilitation process is key for successful outcomes. That is why, upon the first cases of COVID‐19 being reported in Chile and the country entering a long period of quarantine that limited mobilization for several months, the CROFNH saw the need to implement strategies to safely maintain attention of its patients. Chile has reported 51 cases of children with cancer who were infected with COVID‐19; one childdied.^33^

Starting in March 2020, the CROFNH implemented a program of synchronous telerehabilitation, granting individual and integrated videoconferences of 45 to 60 minutes with patients and their families. During these sessions, one or two clinical professionals connected online with the patient, guiding live therapies through the screen. Depending on the therapeutic objective, interactive games were incorporated through the videoconference platform, as well as rounds of exercises or activities that the patient did at home using cushions and different elements, while the professional gave instructions and (as exemplars) performed exercises on the screen.

Because the population served by the CROFNH is mainly from low and middle socioeconomic status, electronic devices (tablets), internet access, and therapeutic equipment (balls, elastic bands, mats, school supplies, among others) were given to the children to facilitate the dual interaction in therapies.

In May 2020, due to the need to continue promoting social skills in our patients, group workshops were initiated in the online version. To date, there have been visits to the zoo, Disney World, the aquarium, communication workshops, and celebrations of Children's Day and Halloween.

As of December 2020, a total of 8549 synchronous telerehabilitation sessions had been granted to 194 different children and adolescents from the following specializations: differential education, physiatry, speech therapy, kinesiology, neuropsychology, nutrition, psychology, and occupational therapy.

Through the above‐described modality, it was also possible to continue monitoring the status of patients in palliative care and accompany the family in the end‐of‐life process without exposing the patient to infection risk. In 2020, the CROFNH gave 1252 rehabilitation sessions to patients in palliative care, of which 1004 sessions occurred through synchronous telerehabilitation.

In many health areas and disciplines, telerehabilitation modalities are showing great potential to secure and/or improve care access and continuation during and after the crisis of COVID‐19. Strokes, post‐surgeries, heart, and musculoskeletal conditions are examples in which telerehabilitation is being used effectively.^34^ In the context of childhood chronic conditions and disabilities, telerehabilitation programs may be beneficial for child and family well‐being as well as for continuity of care.^35^

### 3.1 Benefits, disadvantages, and challenges of telerehabilitation

In addition to the beneficial results in the continuity of the rehabilitation processes for all children, this service showed other advantages: (a) the positive family participation and commitment, (b) the caregivers' capacity of taking the role of co‐therapist, (c) the potential to overcome physical borders (one patient was in Bolivia while receiving our sessions), and (d) the importance of adaptation and innovation capacities of the clinical team.

Among the limitations observed in this modality are the following: the need for a good internet connection that maintains uninterrupted sessions; the impossibility of administering all tests through the screen; and the loss of therapeutic physical contact, which means that certain activities are not carried out for safety reasons (e.g., balance or active swallowing).

## 4 FUTURE CHALLENGES

There are several future challenges for CROFNH: (a) the increase of coverage to other regions of the country, (b) the increase of the range of benefits it provides in both complementary and traditional therapies (orthotics laboratory, neurology, psychiatry, art therapy, music therapy, smile therapy, among others), and (c) the continuation of incorporating innovation (3D printer, new software, and equipment).

Specific to telerehabilitation, there are challenges such as incorporating sensors, software, and tools that allow a reliable measurement, as well as objective monitoring of patients from a distance.

As for challenges related to wider health care coverage, worth mentioning are the search for permanent sustainability of the center, the promotion of increased coverage of health insurance, the promotion of research, and the replication of the model in the rest of Latin America.

We are aware of the need to know and measure clinical results as we keep high‐quality standards of care. That is why we are working on a system of indicators and the systematization of data collection that provides better information of both the individual and the collective impact of the CROFNH work. This data collection can constitute a resourceful database about the rehabilitation situation of childhood cancer in Chile.

## 5 FINAL REFLECTION

Finally, it is important to mention there is still much work to do in order to achieve equity. Currently, not all children and adolescents with cancer have access to a fundamental right such as rehabilitation; not all have the opportunity to become autonomous, independent adults, capable of contributing to our society. It is an urgent need to join efforts so that all children and adolescents with cancer, regardless of their socioeconomic situation, have access to a specialized, comprehensive, and early rehabilitation program that contributes to improve quality of life and social inclusion.

The CROFNH example is in line with the recommendations for models that should provide multidisciplinary, comprehensive childhood cancer care aiming at minimizing short‐ and long‐term cancer morbidity.^36^, ^37^ We show with the CROFNH program that granting this access is necessary and possible, and we must turn to collaboration between governmental, private, and civil society organizations in order to take a leap in the quality of life for future childhood cancer survivors.

## CONFLICT OF INTEREST

The authors have stated explicitly that there are no conflicts of interest in connection with this article.

## ETHICAL STATEMENT

Statistics in this manuscript are based on internal data of the CROFNH and do not involve patient identification or other sensitive information. Therefore, no authorization from the ethics committee or informed consent was required.

## AUTHOR CONTRIBUTIONS

**Vera Celedon:** Conceptualization; data curation; formal analysis; writing ‐ original draft. **Nuria Rossell:** Conceptualization; formal analysis; supervision; writing‐review & editing. **Marcela Zubieta:** Conceptualization; formal analysis; supervision.

## Data Availability

Data sharing is not applicable to this article as no new data were created or analyzed in this study.
